# The Future of the Hospitals

**Published:** 1910-08-06

**Authors:** 


### The Hospital

A Journal of

The Medical Sciences and Hospital Administration.

New Series No. 182, Vol. VII. [No. 1254, Vol. XLVIII.] SATURDAY, AUGUST 6, 1910

### The Future of the Hospitals.

The debate on "The Economic Basis of Hospital Management" at the Medico-Sociological Section of the British Medical Association last week, a full report of which appears elsewhere in this issue, touched upon subjects which, from their inherent importance to all institutional workers, are of more than ordinary interest. Professor Moore, in opening the discussion, laid stress on several points which have hitherto been considered highly debatable, and which will yet be regarded with very mixed feelings by those who esteem our hospital system of to-day as entitled to the respect and admiration which are usually given to things sacrosanct. Yet it does not need Professor Moore's strong special pleading—which, like all indictments against specific evils, errs on the side of strictness, inasmuch as it forgets to take into full account the merits of the system it attacks—to make the thoughtful realise that there is much necessity for reform. Perhaps the very onesidedness of the attack which initiated the debate will frighten some of us who have been inclined to look upon the voluntary system as an eagle mewing her "mighty youth," and who are prone to forget, owing to our ingrained affection for what after all is the finest model of organised charity extant, that there are faults even in that system!

To take the arguments for and against the present condition of affairs would be to indulge in a disquisition far beyond our moderate limits. Still, it is obvious that many of our readers will not read the report without finding food for reflection, and let us add for opposition, in the statements made by the opener. The premiss, for instance, that the hospital fulfils a dual object, and that it is as necessary for purposes of study and training as it is for the care of the sick, is one which will not be readily agreed to by everyone. Again, we should like to know the institutions where, according to Professor Moore (we quote from our report), one resident officer attends to five hundred beds. And we are inclined to think too much is made of the objection that the governing bodies consist largely of lay members, and that when an appointment became vacant (we quote again) there was all the canvassing which was so objectionable. Appointments on the staff are nowadays usually made by the medical committee, and only on rare occasions does the lay board refuse to ratify them. With regard to the question of hospital abuse and the want of co-ordination that at present exists between the various institutions, there is, on the other hand, a fairly definite consensus of opinion. So, too, with regard to the question of payment of staff members, and, as more than one speaker pointed out, it would be in the interests of the hospitals to initiate reforms in these matters. Equally evident is the fact that the present is an opportune moment for commencing a definite scheme of reform that will be effective and satisfactory; the only question is as to the best form that scheme is to take and the manner in which it is to be carried into being.

Sir Henry Burdett, whose experience and lifelong interest in the great problem of hospital management have made him an authority on the subject, and whose views necessarily call for earnest and careful consideration, made a strong appeal for the co-operation of all workers in the field to put the whole subject of medical relief once and for all on a proper and business-like footing. The general principles of his scheme have already been outlined in the address given before the Home Hospitals Association, reported in The Hospital of March 26, and since republished in pamphlet form. With those principles our readers are sufficiently acquainted, and we take it that they will agree, upon studying all the aspects of the question, that if the scheme could be carried into effect it will result in the establishment of a uniform system that will be ideal in many respects, and which will certainly mean a thorough reform of existing conditions. And yet it is disheartening to find that some at least of the speakers in last week's discussion went off into side issues and expatiated upon details rather than attempted to deal with the broad outlines of the scheme. It raises the important question whether members of the profession, even those who take an interest in medico-sociological questions, are so thoroughly acquainted with hospital management and with the vital problems presented by our present system as to be able to discuss any proposed alterations. No man can lightly consent to any modification of a system unless he fully realises the inevitable consequences which a measure of reform calculated to bring about such modification will produce. In order to voice an honest and well-considered opinion on the problem it is necessary that those who take part in the discussion shall have compared the hospital system as it exists in this country with other systems which obtain elsewhere. To look at the question of hospital reform merely from the point of view of the general practitioner is to lose sight altogether of the many other, and in our opinion far more important, issues involved. And it is manifest that the majority of those who followed in the wake of the two principal speakers found it impossible to get rid of the prejudice of professionalism and to grapple with the main issues in a broad and statesmanlike manner.

The discussion has brought the whole matter of hospital reform prominently before the profession. The subject with which it dealt can no longer be considered, as it was by some members of the profession until very recently, as in the nature of *questiones quodlibeticæ*. The Poor Law Commission Reports and the general feeling that the present system is not all that could be wished, have profoundly influenced us during the past twelve months, and it is a sign of the times that the British Medical Association has thought fit to give prominence to a matter so eminently topical. All that it is allowable to conclude from the debate is that there exists a desire on the part of the medical profession to deal with the question in a serious manner. It now remains to materialise that desire into action, by joining hands with the institutional workers whose thoughts have already long since been centred round the subject of reform, to bring about a reconstituted medical service which, as the discussion proved, will be heartily welcomed by the profession at large.

